# Anterior Subluxation after Total Hip Replacement Confirmed by Radiographs: Report of Two Cases

**DOI:** 10.4061/2011/519254

**Published:** 2011-05-10

**Authors:** Conor P. McGrory, Brian J. McGrory

**Affiliations:** ^1^Maine Joint Replacement Institute, Portland, ME 04102, USA; ^2^Orthopaedic Associates, 33 Sewall Street, Portland, ME 04104, USA; ^3^Joint Replacement Center, Maine Medical Center, Portland, ME 04102, USA; ^4^Division of Orthopaedics and Rehabilitation, College of Medicine, The University of Vermont, Burlington, VT 05405, USA

## Abstract

Demonstrable anterior subluxation of the femoral head after a total hip arthroplasty is a rare complication and is usually transient. Both a case of recurrent subluxation and a case of chronic subluxation are described in this paper, each one presenting with unexpected femoral head eccentricity in the acetabulum on radiograph. We show how this unusual complication can be successfully identified and treated.

## 1. Introduction

Instability after total hip replacement (THA) occurs infrequently and leads in most cases to hip dislocation. In rare cases, instability presents as transient subluxation [1–3]. A patient reports a sound or click and often has a sense of giving way or apprehension. Dislocation and subluxation of the hip after THA normally occur in the first three postoperative months, but cases of late instability are not uncommon [4, 5].

Obvious polyethylene wear is a familiar radiographic finding after noncontemporary THA, but is very uncommon in the first decade when cross-linked polyethylene is used. On the other hand, early eccentric head position may signify fracture of the polyethylene, which is more common after the material changes brought on by the cross-linking process [6, 7]. Polyethylene wear and subluxation are related because not only can instability cause polyethylene wear, but polyethylene wear can cause subluxation or dislocation [8, 9]. Polyethylene wear [10], cross-linked polyethylene fracture [7, 11], and THA instability [12] are all more common in the setting of acetabular component malposition (especially increased abduction).

We present two case examples where late anterior THA subluxation presented as unexpected femoral head eccentricity in the acetabulum. We discuss how this unusual complication can be successfully identified and treated.

## 2. Case Examples

We reviewed two cases of patients who presented with apparent eccentric polyethylene wear and pain, after contemporary THA. In both situations the acetabular component was positioned in excessive abduction. After direct lateral radiograph confirmed femoral head subluxation and examination under fluoroscopy with the leg internally rotated confirmed reduction, revision surgery was offered. Both patients underwent revision surgery to reposition the cup, and in both cases pain-free ambulation, without recurrent subluxation, was achieved after recovery.

### 2.1. Patient 1

**Figure 1:**
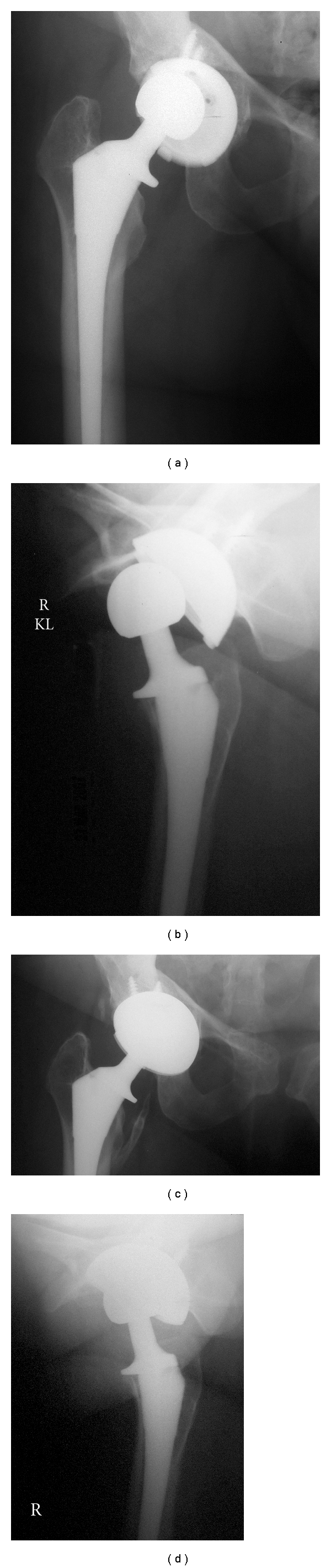
(a) Patient 1: anteroposterior radiographs demonstrating eccentric placement of the femoral head within the acetabular polyethylene and excessive acetabular component abduction. (b) Patient 1: direct lateral radiograph demonstrating anterior femoral head subluxation and excessive acetabular component anteversion. (c) Patient 1: anteroposterior radiographs demonstrating concentric femoral head positioning and corrected acetabular component abduction. (d) Patient 1: direct lateral radiograph demonstrating concentric femoral head positioning and corrected acetabular component abduction.

A 56-year-old female presented with groin pain and leg-length inequality one year after a right, noncemented total hip arthroplasty (THA) performed for osteoarthritis through a posterior approach. Review of the implants used at the time of surgery confirmed that the femoral head size correctly matched the acetabular inner diameter size. Physical examination demonstrated an antalgic gait to right side, mild pain over greater trochanter and the right leg being about 0.5 cm longer than the left. Supine radiographs showed eccentric positioning of the femoral head within the acetabular polyethylene, and excessive acetabular component abduction (Figure 1(a)). Direct lateral radiograph confirmed femoral head subluxation (Figure 1(b)) and excessive acetabular component anteversion. Examination under fluoroscopy with the leg internally rotated confirmed reduction and ruled out catastrophic polyethylene failure/fracture. Of note, review of immediate postoperative radiographs confirmed that the component position had not changed since original implantation.

Revision surgery was offered, and at the time of surgery, examination confirmed chronic subluxation of the femoral head anteriorly. There was no obvious polyethylene wear of the component, and there was no plastic fracture. There was evidence of posterior femoral neck-on-polyethylene impingement. The femoral head remained partially in the acetabulum because of a very thick anterior hip capsule. The femoral component was not malpositioned and was well fixed. The acetabular component was revised to a more acceptable position. Confirmation of appropriate positioning was made by intraoperative range of motion testing and anteroposterior pelvis radiograph.

Postoperatively, the patient was allowed full weight-bearing and a brace was not used. There were no known complications. She had no further pain and no signs or symptoms of instability. One-year follow-up radiograph showed no further subluxation (Figures 1(c) and 1(d)).

### 2.2. Patient 2

**Figure 2:**
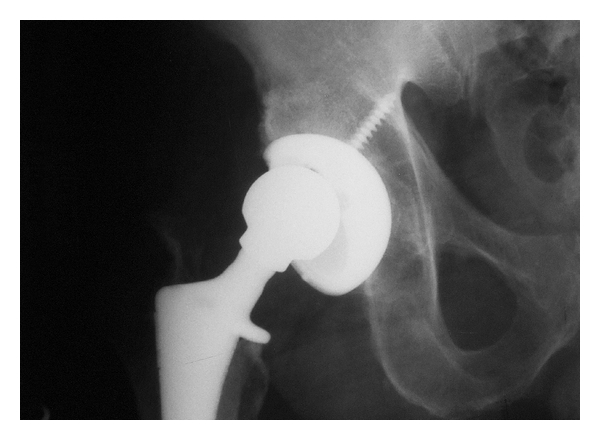
Patient 2: anteroposterior radiographs demonstrating eccentric placement of the femoral head within the acetabular polyethylene and excessive acetabular component abduction.

A 64-year-old male presented with generalized hip pain and associated clicking five years after a right, noncemented total hip arthroplasty (THA) performed for osteoarthritis through a posterior approach. Symptoms were present for 6 months and were not associated with trauma. Review of the implants used at the time of surgery confirmed that the femoral head size correctly matched the acetabular inner diameter size. Physical examination demonstrated an antalgic gait to right side, and the patient used a crutch on his left side. There was an audible clunk with ambulation, but this could not be reproduced on physical exam. Supine radiographs showed superior eccentric placement of the femoral head within the acetabular polyethylene and excessive acetabular component abduction (Figure 2). Prior radiographs were not available for comparison. Radiographs in internal rotation showed the head to be concentrically reduced, and revision surgery was offered.

At the time of surgery, examination confirmed acetabular malposition in excessive abduction and anteversion. There was no obvious polyethylene wear of the component, and there was no plastic fracture. There was evidence of posterior femoral neck-on-polyethylene impingement. The femoral component was not malpositioned and was well fixed. The acetabular component was revised and the femoral head size increased to 36 mm. 

Postoperatively, the patient was allowed full weight-bearing and a brace was not used. There were no known complications. He had no further pain, and no signs or symptoms of instability. Three month follow-up radiograph showed no further subluxation.

## 3. Discussion

This is the first report, to our knowledge, that documents the radiographic presentation and treatment of both chronic (patient 1) and intermittent (patient 2) anterior THA subluxations. 

The radiographic presentation of this complication mimicks polyethylene wear, a much more common finding after THA. In this era of ubiquitous cross-linked polyethylene usage, early wear of this type is rarely if ever seen, but catastrophic failure such as polyethylene fracture needs to be considered. “Run-away” wear and polyethylene fracture are both found more commonly with acetabular component malposition in excessive abduction. When a patient presents with an apparent eccentric placement of the femoral head in the acetabulum, the differential diagnosis includes bearing surface size mismatch, polyethylene wear, polyethylene rim fracture, and femoral head subluxation. 

Implant size mismatch of the femoral head and polyethylene inner diameter may present with the femoral head in a superior eccentric position if the head is smaller than the acetabulum. This diagnosis can be ruled out by reviewing the implant sizes from the hospital chart and operative notes.

Polyethylene wear that is visible to the naked eye is common in older prostheses with early generation polyethylenes. This is often associated with periprosthetic osteolysis and is noted to progress gradually over time. Confirmation of contemporary polyethylene (again by review of hospital records) and sudden, dramatic changes in apparent wear make this diagnosis unlikely.

Rim fracture can be seen in older polyethleyne components, with prior wear and poor design, as well as newer cross-linked polyethylene. Risk factors for fracture include thin components, particularly at the locking mechanism, association with a large femoral heads, and vertical cup placement (excessive abduction).

Instability of the femoral head occurs because of hypermobility of the joint and/or malpositioning of the prosthesis. The direct lateral radiograph or fluoroscopic examination with internal rotation of the femur confirms the diagnosis of anterior THA subluxation. Once the problem of a subluxating femoral head has been identified, revision surgery is often necessary. In most cases involving late instability of the hip, surgery is often needed to reposition the components, which have already healed into place. The surgeon needs to remove and reposition the components that are malpositioned, which in the cases presented here was limited to the acetabular side. In both case reports that we reviewed, this treatment worked very well.

We are not certain why the patients presented did not dislocate their total hip replacements. Immediate postoperative radiographs in case 1 demonstrated that the components did not change to become malpositioned; case 2 radiographs were not available. One possibility is that the patients' hips were protected from dislocation by “hip precautions” after surgery. After the capsule had healed, relaxation of precautions and subsequent increased motion lead to subluxations. Once the soft tissues were healed anteriorly, dislocation was less likely. 

In conclusion, we show two cases of late onset THA instability: one was manifest by chronic and the other by intermittent anterior-superior subluxation of the femoral head. The presenting radiographs showed apparent eccentric head placement within the acetabulum which could be due to component mismatch, polyethylene wear, polyethylene fracture, or subluxation. After confirming that the components are not mismatched, direct lateral radiograph or fluoroscopic examination with internal rotation verifies the diagnosis of femoral head subluxation and reduction. If the acetabular component is malpositioned in excessive anteversion or abduction (as it was in both of these cases), revision surgery is indicated and successful in resolving the problem.

##  Ethics Committee Approval

The corresponding author confirms that any research associated with this manuscript underwent ethics committee approval and the correct procedure was followed regarding releases and ethical research standards.
